# How compassionate communities are implemented and evaluated in practice: a scoping review

**DOI:** 10.1186/s12904-022-01021-3

**Published:** 2022-07-20

**Authors:** Katia Dumont, Isabelle Marcoux, Émilie Warren, Farah Alem, Bea Alvar, Gwenvaël Ballu, Anitra Bostock, S. Robin Cohen, Serge Daneault, Véronique Dubé, Janie Houle, Asma Minyaoui, Ghislaine Rouly, Dale Weil, Allan Kellehear, Antoine Boivin

**Affiliations:** 1 Canada Research Chair in Partnership With Patients and Communities, University of Montreal Hospital Research Center (CRCHUM), Pavillon S, 850 Saint-Denis Street, Montreal, QC S01.136H2X 0A0 Canada; 2 Faculty of Health Sciences, University of Ottawa, 25 University Street, office 206, Ottawa, K1N 6N5 Canada; 3 Montreal Palliative Care Institute, 265 André-Brunet Street, Kirkland, QC H9H 3R4 Canada; 4 Departments of Oncology and Medicine, Faculty of Medicine and Health Sciences, McGill University, Montreal, Canada; 5 Lady Davis Research Institute of the Jewish General Hospital, Palliative Care Research, room E8.06, 3755 Côte Ste. Catherine Road, Montreal, QC H3T 1E2 Canada; 6 Department of Family Medicine and Emergency Medicine, Faculty of Medicine, Université de Montréal, 2900 Édouard-Montpetit Boulevard, Montreal, QC H3T 1J4 Canada; 7 Centre de Recherche de L’Institut Universitaire de Gériatrie de Montréal, 4565 Chemin Queen-Mary, , Montreal, QC H3W 1W5 Canada; 8 Marguerite d’Youville Research Chair On Humanistic Nursing Interventions, Faculty of Nursing, Centre-Ville Station, Université de Montréal, P.O Box 6128, Montreal, QC H3C 3J7 Canada; 9 Department of Psychology, Université du Québec À Montréal, 100 Sherbrooke Street West, Montreal, QC H2X 3P2 Canada; 10 College of Nursing and Health Sciences, University of Vermont, Burlington, USA

**Keywords:** Compassionate communities, Implementation, Evaluation, Palliative care, End of life, Health promotion

## Abstract

**Background:**

Compassionate communities are rooted in a health promotion approach to palliative care, aiming to support solidarity among community members at the end of life. Hundreds of compassionate communities have been developed internationally in recent years. However, it remains unknown how their implementation on the ground aligns with core strategies of health promotion. The aim of this review is to describe the practical implementation and evaluation of compassionate communities.

**Methods:**

We undertook a scoping review of the empirical peer-reviewed literature on compassionate communities. Bibliographic searches in five databases were developed with information specialists. We included studies in English describing health promotion activities applied to end-of-life and palliative care. Qualitative analysis used inductive and deductive strategies based on existing frameworks for categorization of health promotion activities, barriers and facilitators for implementation and evaluation measures. A participatory research approach with community partners was used to design the review and interpret its findings.

**Results:**

Sixty-three articles were included for analysis. 74.6% were published after 2011. Health services organizations and providers are most often engaged as compassionate community leaders, with community members mainly engaged as target users. Adaptation to local culture and social context is the most frequently reported barrier for implementation, with support and external factors mostly reported as facilitators. Early stages of compassionate community development are rarely reported in the literature (stakeholder mobilization, needs assessment, priority-setting). Health promotion strategies tend to focus on the development of personal skills, mainly through the use of education and awareness programs. Few activities focused on strengthening community action and building healthy public policies. Evaluation was reported in 30% of articles, 88% of evaluation being analyzed at the individual level, as opposed to community processes and outcomes.

**Conclusions:**

The empirical literature on compassionate communities demonstrates a wide variety of health promotion practices. Much international experience has been developed in education and awareness programs on death and dying. Health promotion strategies based on community strengthening and policies need to be consolidated. Future research should pay attention to community-led initiatives and evaluations that may not be currently reported in the peer-review literature.

**Supplementary Information:**

The online version contains supplementary material available at 10.1186/s12904-022-01021-3.

## Background

The concept of compassionate community is rooted in a health promotion approach to palliative care [1, 2]. Historically, the vision of compassionate communities has been anchored in the work of the World Health Organization’s Ottawa Charter [3] and Healthy City movement [4, 5]. The Ottawa Charter describes five major pillars of health promotion: *1- Develop personal skills, 2- Create supportive environment, 3- Reorient health services, 4- Strengthen community actions, 5- Build healthy public policy*. Kellehear adapted these core health promotion strategies to palliative care, proposing that “the goals of health-promoting palliative care are to provide education, information and policy-making for health, dying and death.” ([5] p. 26).

Compassionate communities address a holistic definition of health that goes beyond the simple treatment of symptoms to include psychological, spiritual and social well-being. It emphasizes the strengthening of social capital, mutual aid relationships and the ability of citizens to actively participate in the development of their communities, to create social connections and care for each other [6]. As an intersectoral approach, compassionate communities mobilize a variety of civic actors outside the professional health care system (e.g., education, municipalities, community organizations, spiritual groups), as well as patients, family members, neighbors and citizens. As a model, it encourages partnership building to support community capacity-building and resilience in issues surrounding dying, death and bereavement, often with a focus on health inequalities, diversity and social inclusion.

Health promotion approaches have received considerable attention within and beyond palliative care. In recent decades, hundreds of compassionate communities have been established around the world [7]. This demonstrates an international willingness towards community-led models of social and practical support for people living with advanced illness and their caregivers ([8] p.1). However, it remains unknown how the implementation of compassionate communities on the ground aligns with core principles for health promoting palliative care.

The aim of this review is to describe the practical implementation of compassionate communities, analyze which stakeholders are engaged in their development, what core health promotion strategies are used, and how compassionate communities are evaluated.

### Review questions

The goal of this review was to understand how compassionate communities have been implemented and evaluated in practice. More specifically, we sought to describe:

1. Which stakeholders are engaged in compassionate community development?
2. What barriers and facilitators have been identified?
3. Which health promotion activities have been implemented in practice?
4. How have compassionate communities been evaluated?

## Methods

Given the diversity of the literature on compassionate communities, we adopted a scoping review design as a preliminary evaluation of available research literature [9]. As described by the Joanna Briggs Institute [10], scoping reviews are best suited “to answer questions regarding the nature and diversity of the evidence/knowledge available” (p. 409). Our review process was composed of 5 steps: 1- identification of potential studies; 2- sorting references and selecting studies; 3- data extraction; 4- analysis and synthesis; and 5- collating, summarizing, and reporting the results to inform practice and future research. The review protocol is available from the authors upon request. Reporting of the scoping review methods and results followed PRISMA-ScR reporting guidelines (Additional file 5) [11].

### Search strategy

We designed, piloted and implemented our bibliographic search strategies with the help of information specialists. The search strategy was limited to English language articles published but without restrictions regarding the year of publication. Initial search terms were built around the following concepts: (end-of-life, dying, palliative care) AND (compassionate communities, compassionate cities OR health promotion). A pilot search was undertaken on *Ovid MEDLINE* and *EBSCOhost-CINAHL* to refine the search strategy, using previously identified articles meeting our inclusion criteria. Text words and index terms were adapted, based on this preliminary search. A final, more detailed search was conducted in August 2019 in *EBSCOhost-CINAHL, EMBASE, Ovid MEDLINE, Ovid PsychInfo, Web of Science.* The final search strategy, and its adaptation for specific electronic databases is described in Additional file 1: “ Search Strategy. Research results were saved using the bibliographic management software EndNote.

### Inclusion and exclusion criteria

To be included, published articles needed to: 1- be an empirical article; 2- describe health promotion activities, as defined by the World Health Organization Ottawa Charter for Health Promotion [3]; 3- be applied to end-of-life or palliative care, including care of bereaved people.

Abstract-only publications (i.e., no full-text article available), grey literature, books and doctoral theses were excluded. Only empirical studies and articles in journals were included, conceptual or theoretical papers, advocacy, opinion articles and secondary literature were excluded. Published systematic reviews were not analyzed directly but were used to identify further primary studies and articles meeting our inclusion criteria. Three research assistants applied the inclusion and exclusion criteria of identified abstracts and articles, without automatic computer screening assistance. Disagreements were resolved through discussion, or in consultation with the two study principal investigators.

### Analysis and presentation of results

All included papers were imported into QSR International’s NVivo 12 software for data extraction and analysis. Included articles were imported directly into NVivo for data extraction and qualitative thematic analysis. Analysis proceeded in two stages. In Stage 1, we used an inductive approach to categorize the stakeholders involved, barriers/facilitators, and activities evaluation approaches (study design, and evaluation measures). In Stage 2, we used a deductive approach to organize the presentation of results, according to existing frameworks and models for barriers and facilitators [12], health promotion activities (Ottawa Charter), and categories of evaluation measures [13]. To analyze how compassionate communities were evaluated, we only analyzed articles that reported both their evaluation methods and results within the article (i.e., we excluded from this analysis articles reporting “evaluation results” without mentioning how these evaluations were conducted). We classified study design based on Hartling’s [14] design classification tool.

### Community engagement in research

To increase the pragmatic value and relevance of the review, community members and practitioners were engaged in the design of the scoping review protocol, interpretation of findings, and co-authorship of the manuscript. Following a collaborative participatory research approach [15], community members and practitioners (patient partner, community developer, healthcare professional and palliative care manager) were integrated in the scoping review steering committee, which oversaw the design (questions and scope) of the review, participated in a two-hour data interpretation workshop to analyze implications of findings for practice, and contributed as co-authors on the manuscript to increase relevance for practitioners.

## Results

**Fig. 1:**
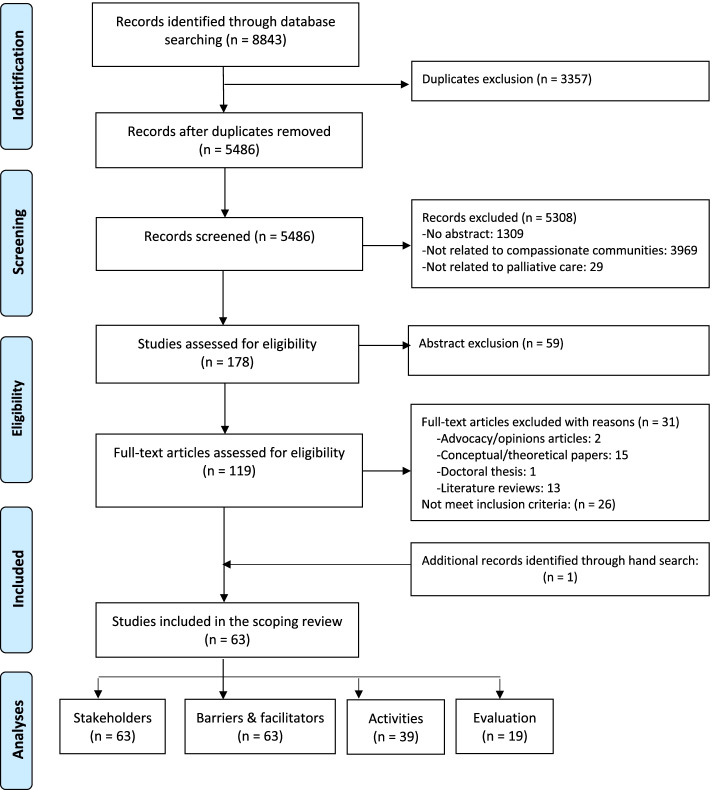
Flow chart of included studies

Figure 1 provides a PRISMA flow chart diagram of included studies [16]. A total of 5486 individual records were screened for eligibility and a final set of sixty-three studies (n = 63) were included for analysis. Of the 63 articles included in the scoping review, all (100%) included information on stakeholder engagement as well as barriers and facilitators for implementation. Of the included papers, 39 (62%) provided information about the type of health promotion activities implemented, and 19 (30%) provided information about evaluation methods and results.

### Description of included studies

Table 1 provides a summary description of included studies (*n* = 63). Most included articles were published after 2011 (74.6%). The majority of studies were conducted in the United States (USA) (30.6%), Australia (28.6%), and the United Kingdom (UK) (15.9%) and with a minority of articles (4.8%) from developing countries (Malaysia, India and Uganda). The detail of all 63 included articles is provided in Additional file 2: Description of included articles.

**Table 1:** Description of included articles

**Included articles (*N* = 63)**		**Number of articles**	**Percent (%)**
Publication year	Before 2000	3	4.8
	2001–2010	13	20.6
	2011-present	47	74.6
Country	USA	19	30.2
	Australia	18	28.6
	UK	10	15.9
	Canada	5	7.9
	Austria	2	3.2
	India	2	3.2
	Spain	2	3.2
	Sweden	2	3.2
	Ireland	1	1.6
	Malaysia	1	1.6
	Portugal	1	1.6
	Scotland	1	1.6
	Uganda	1	1.6

### Which stakeholders are engaged in compassionate community development?

The included literature reports on a variety of stakeholders engaged in compassionate communities’ development. Table 2 classifies these stakeholders by individual and organizational categories. Health and social care providers (55.5%) and health services (44.4%) are most often engaged as compassionate community stakeholders and leaders. Community members were engaged in 47.6% of articles, most often as target population and participants (42.8%), in contrast to being the leaders and co-leaders (20.6%) of compassionate community initiatives. Most of the published programs target the general population (people at the end of life, caregivers and bereaved people), with a minority of included articles specifically reporting on programs designed with and for marginalized populations (eg. ethnic minorities).

**Table 2:** Stakeholders engaged in compassionate communities’ development

**Individual stakeholders**	**Description and examples**	N*	**%**
Health and social care providers	Nurses, palliative care workers, physicians, social workers	35	55.5
Community members	Members of the public, citizens, general population, community members, children, neighbors	30	46.6
Patients-families-friends		28	44.4
Volunteers		24	38.1
Leaders-administrators	Coordinators (bereavement, community program), administrators (health care, tribal health, institutional), leaders (administrative, public policy), funeral home directors	20	31.7
Other civic actors	Thanatologists, artists, attorneys	18	28.6
Workers	Colleagues, staff, employees	13	20.6
Religious	Priests, spiritual leaders, pastors	12	19.0
Educators & students	Teachers, pupils	10	15.9
Researchers		6	9.5
**Organizational stakeholders**	**Description and examples**	**N**	**%**
Health services	Hospices, hospitals, foundations, World Health Organization, palliative care associations	28	44.4
Education	Universities, schools	15	23.8
Other civic organizations	Prisons, media, libraries, non-profits, foundations	14	22.2
Community groups	Support group, civic associations and committees	11	17.5
Religious organizations	Churches, parishes	11	17.5
Governments	Local government, state, municipality	9	14.3
Businesses	Pharmacies, funeral services	6	9.5

^*^N = number of articles

### Barriers and facilitators for compassionate community implementation

Of the included studies, 13 categories of factors were identified as barriers and facilitators for compassionate community implementation [12] (Table 3). Adaptation to local culture, social attitudes and local context was the most frequently reported barrier for implementation (31.7% of articles), including: social attitudes toward asking for and receiving help at the end of life, alignment of proposed activities with cultural norms and attitudes, and sensitivities to language, religious attitudes and reaching out toward ethnic minorities. Information, open discussions and awareness about the end of life was the most frequently reported facilitator for implementation of compassionate communities (26.0% of articles). Access to informal and political support were also highlighted as key facilitators, along with practical support for traveling, duration of activities, location and convenient scheduling of activities (22.2% of articles for both categories). Styles of leadership facilitating community partnership and empowerment were also highlighted as facilitators. While the availability or lack of human and financial resources may either be reported as a facilitator (20.6%) or barrier (14.3%), existing policies and organizational structures were essentially reported as a barrier for implementation of compassionate communities (15.9% listing it as a barrier, while no article described the role of supporting policies as facilitating implementation).

**Table 3:** Barriers and facilitators for compassionate communities’ implementation

**Factors**	**Description**	**Barriers** N*	**%**	**Facilitators** N*	**%**
**Attitudes**					
Cultural, religious, social	Social attitudes to receiving help, alignment of activities with cultural attitudes, differences (in cultural patterns, perceptions, roles, language), cultural and ethnic diversity	20	31.7	8	12.7
Support	Amount of support, type (ex. emotional, practical, medical), sources (ex. professional), formal/informal, political implication level	8	12.7	14	22.2
Collaboration & partnerships	Working together, sharing vision and mission, negotiating, relation between formal and informal networks, level of connections and commitment	8	12.7	12	12.0
Expectations	Assess and meeting: expectations, visions, needs, wishes of the patients, caregivers, communities, people	6	9.5	12	19.0
Interventions	Level of listening and communication, speaking quality, initiation of discussions opening, quality of community-based interventions design quality, caregiving practices combination, integrity and dedication acting	5	7.9	9	14.3
Leadership	Provide leadership not ownership, community empowerment, combined leadership, shared partnership	0	0	10	15.9
	**Knowledge**				
Information, awareness, promotion	Media reports and relations, level of awareness about the concept of end-of-life or palliative care, degree of knowledge (of the grieving process, etc.), awareness (of social roles, care needs, compassion, etc.), education program	6	9.5	17	26.0
Training, competencies, stakeholders experience	Training quality, competencies and experience levels	14	22.2	0	0
	**External factors**				
Location & timing	Spaces, site, setting (ex. home), travel/access (geographic and demographic context) technical implications, timing (for support), duration (of the support)	8	1.6	14	22.2
Finance	Amount of funding, financial implications, public financing, grants (quantity and continuity)	7	11.1	6	9.5
Resources	Availability and sustainability of resources, informal network, approach type, human resources	9	14.3	13	20.6
Policies, guidance, bureaucracy	Clarity of rules, organizing structure, level of coordination	10	15.9	0	0
Project (organization, development, implementation, evaluation)	Participation, identification, integration, interests, involvement, recruitment (leaders, volunteers and clients), project definition and tangibility, evaluation (of the program, results, methodologies), clarity of objectives, professional structures	8	12.7	11	17.5

^*^N = number of articles

### Which activities have been implemented in practice?

**Fig. 2:**
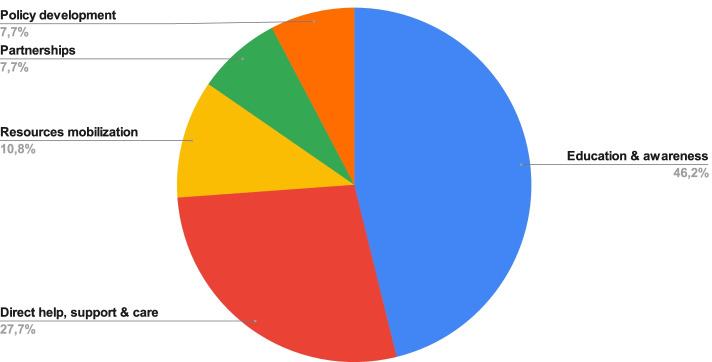
Compassionate community activities

Figure 2 organizes activities according to compassionate community development stages. Needs assessments: includes activities related to exploring existing interventions, assessing community capacity and identifying needs. These activities include focus groups, interviews, literature review, consultations, etc. Needs assessments activities represented only 9.4% of compassionate community development activities reported in the included literature. Stakeholders’ engagement & mobilization: includes activities related to solicitation, recruitment and training of stakeholders involved in the implementation of the design and implementation of compassionate communities. These represented 13.1% of all reported activities. Priority setting: includes activities related to planning activities and agenda setting, framing of the problem and target population, and prioritization of activities and resources. This phase represented only 6.1% of the overall identified activities reported in the literature, which is the less represented category.

**Fig. 3:**
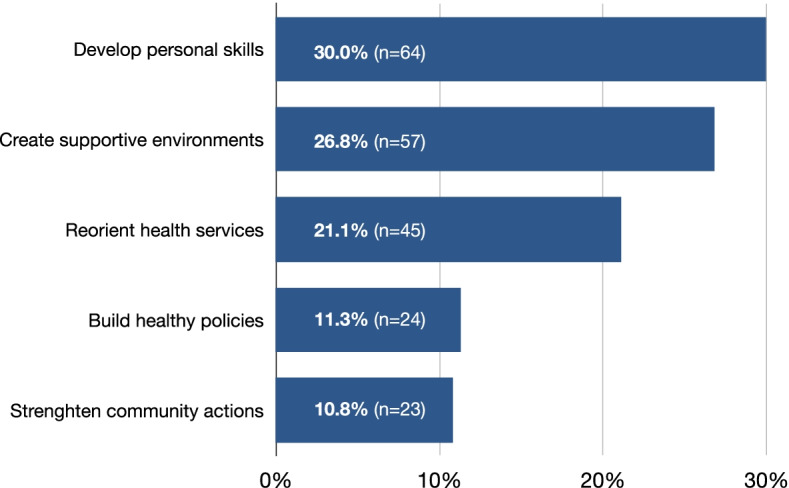
Classification of health promotion strategies.Legend: *n* = *number of activities*

Implementation: this stage includes a description of compassionate communities’ activities implemented in practice. Description of implemented activities represented the most commonly reported stage of development for compassionate communities (64.3% of reported activities), as opposed to upstream development stages (needs assessment, priority setting and stakeholder engagement) and evaluation.

Implemented activities were further categorized inductively (Fig. 2). In order of frequency, these activities included: education and awareness programs (46.2%); direct help, support, and care (27.7%); resources mobilization and linkages (10.8%); build partnerships and collaborations (7.7%); policy development and lobbying (7.7%). Table 4 offers specific examples for these activities. A complete list of activities described in the included article is provided in Additional file 3 “*List of activities”*.

**Table 4:** Examples of activities

**Types of activity** (*n* = nb of activities; %)	**Specific illustrative examples**
**Education and awareness**	Trainings: for health professionals, volunteers, caregivers, faith communities, solicitors (to help them discuss death and dying issues with their clients when drawing up wills and advance care planning
	Health awareness campaign consisted of skit, pamphlet distribution, poster presentation, giving door-to-door information, and general interaction with palliative team in the village
	Workshops & conferences for health professionals, public policy leaders, public
	Camp (activities for children, education and interactive session about death and loss)
	Publications, video and printed materials
	Website creation
	Encourage TV and radio coverage promoting the choice to die at home
	Exhibition and drop-in stands at large events, libraries, places of worship, social/cultural events
	Community Group session in community settings, grief education (in senior housing, churches, assisted living facilities, and businesses)
**Direct help, support and care**	Supported churches to expand outreach programs
	Café Conversation
	Psychology students counseling of bereaved people: a partnership with the university
	Lead from behind—enable others through coaching, mentoring and encouragement
**Resource’s mobilization and linkages**	Sharing of individual and community resources
	Development and diffusion of pain management resources
	Publishing a lighthearted, illustrated trade book and website/blog to make a difficult topic palatable and engaging to a broad audience
**Build partnerships and collaborations**	Broker interagency agreement for collaboration for care delivery
	Building community relationships, external linkages
	Implement memorandums of understandings with external service providers
	Projects in partnership with schools, aged care facilities and groups, community health services, service clubs, faith communities, local government and neighborhood houses were among the community services and groups
**Policy development and lobbying**	Create policy documents to guide funders and program planners
	Propose fiscal policies to reorient healthcare services for dying, death, loss, and bereavement
	Lobby research organizations to prioritize end-of-life research, including community-based participatory studies
	Promote lobbying by HIV-positive people in collaboration with hospices for development of specific HIV policies
	Insert healthy end of-life principles into existing and new policies alike, and remove unhelpful policies that undermine good outcomes in end-of-life care. Policy settings include local government, community health services, primary health and medical practitioners and community service organizations

Figure 3 offers a complementary perspective on activities implemented by compassionate communities, categorized according to the five Ottawa Charter action strategies for health promotion. The most frequent action strategy was aimed at developing personal skills of community members (30%), while two pillars of health promotion were less frequently targeted: building health policy (11.3%) and strengthening of community actions (10.8%).

### How have compassionate communities been evaluated?

**Fig. 4:**
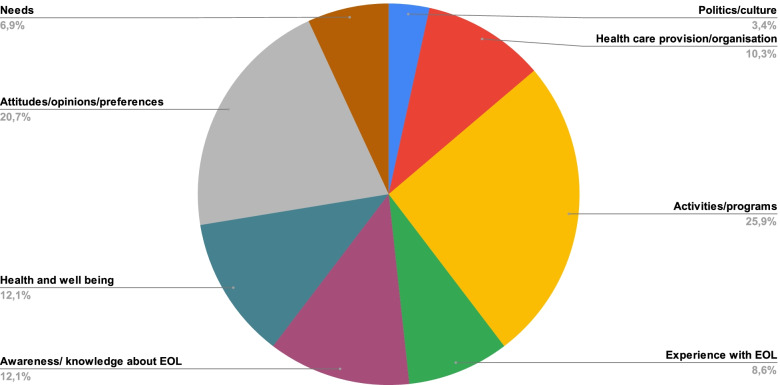
What is being measured in evaluation. Legend: *n* = number of evaluation indicators. *EOL* = End-of-life

*Evaluation* constituted 6.6% of compassionate communities’ activities. The goals of evaluation reported in the articles include: 1- research (for example: verification of achievements [17], 2- quality improvement (example: evaluation of practice innovations with key stakeholders [18]) and 3- certification (as a compassionate community, institution or city [17]). Figure 4 illustrates what is being measured by compassionate community evaluations. Documentation of process indicators related to *activities and programs* was most frequently evaluated (25.9%), followed by participants’ *attitudes, opinions and preferences* (20.7%). Needs, policies and culture were least often included as part of compassionate communities’ evaluations (9.5%). Additional file 4 includes a detailed description of evaluation approaches (what is measured, how and at which levels) for each article reporting evaluation methods and results.

**Fig. 5:**
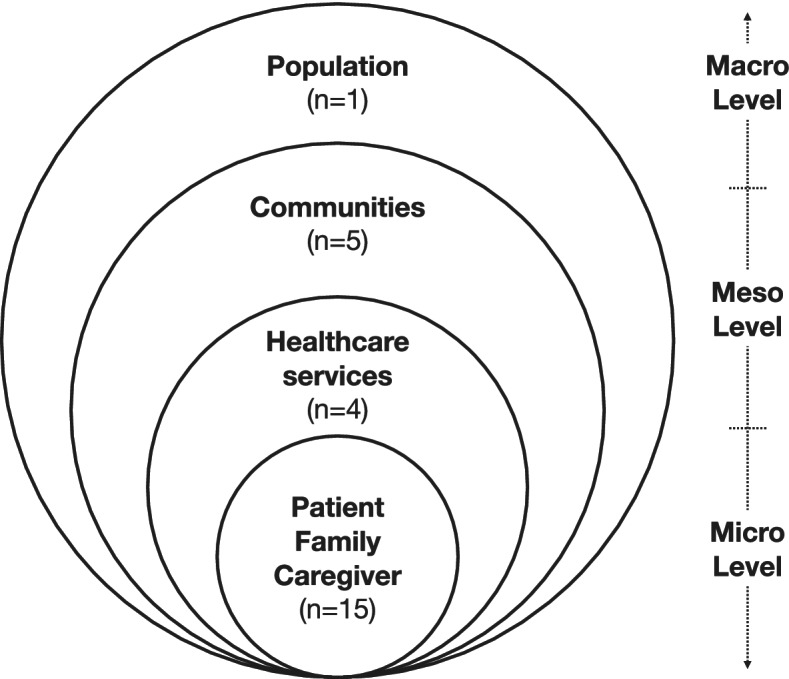
Levels of evaluations. Legend: *n* = number of articles

As health promotion strategies targeting change at the level of communities and populations, compassionate communities processes and outcomes can be collected and analyzed at different levels [13, 19]. Figure 5 classifies compassionate community evaluation measures reported in the included literature. Measurement and analysis at the individual patient, family and caregiver level was most common (79% of articles), followed by meso-level measures of changes in healthcare services (26%) and communities (21%). Macro-level changes were evaluated in only one population study (5%).

**Fig. 6:**
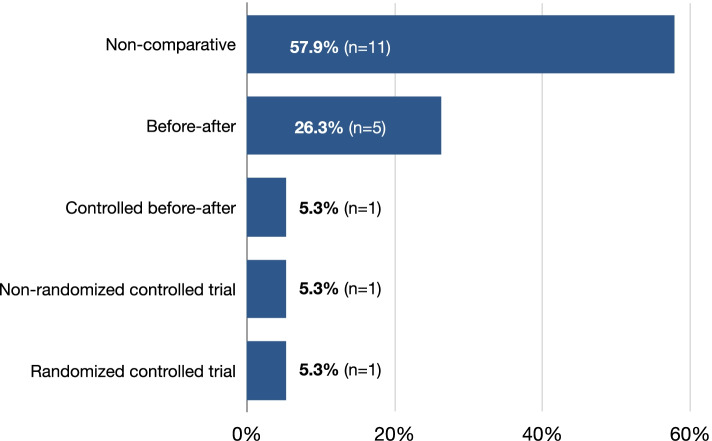
Study design classification. Legend: *n* = number of articles

Based on Hartling’s [14] study design classification tool (Fig. 6), we note that a majority of articles (57.9%) used non-comparative study methods, a few (26.3%) used a before-after study design and one (5.3%) used a controlled before-after study design. Two published protocols of ongoing studies have used controlled trial designs.

## Discussion

This review provides a comprehensive mapping of how compassionate communities have been implemented and evaluated in practice, as reported in the international peer-review literature. A striking finding of this empirical overview is that the umbrella concept of “compassionate communities” encompasses a wide diversity of health promotion practices in the context of palliative and end-of-life and palliative care.

All of the 5 core health promotion strategies described in the Ottawa Charter have been reported in compassionate community initiatives around the world. However, not all strategies are equally represented in practice. Developing personal skills through education and awareness programs represents the majority of reported activities. This finding is consistent with the observation that patients, families and community members are most often engaged as the target audience of compassionate community initiatives, rather than as full partners of community-led programs. Despite the emphasis of ecological approaches to community development highlighted in theoretical models [2, 4, 6], strengthening community action and building healthy public policy are the least common health promotion strategies observed in this review.

Kellehear ([5] p.35) underscores the tendency for health-promoting palliative care to favor direct-services approaches, which aligns with the findings of this review. The fact that most of the reported initiatives are led by healthcare organizations and providers, who may be more accustomed to playing an educator rather than a community facilitator role, may partly explain this dominance of education activities. Alternatively, it is possible that individual education programs serve as a stepping stone for healthcare organizations to initiate collaborations with community groups, or as a pre-condition to put death and dying on the policy agenda. Understanding why and how compassionate community initiatives can move from individual education to community action would be an important area for future development.

Finally, it may also be the case that the neighborhood/local community emphases of the compassionate community movement has led to a more necessary prioritizing of personal skills and creating supportive environments because these seem more relevant to local efforts. The compassionate cities movement, whose emphasis has been similarly ecological in approach, has tended to focus on inter-sectoral co-operation, civic events, policies, and actions across large geographical and cross-institutional terrain (compassionate cities) [5]. While compassionate cities were included in our search strategy, there are far fewer compassionate cities than compassionate communities worldwide and most of them do not have published evaluation. For example, the Public Health Palliative Care International Association website currently lists only 10 cities aspiring to use health-promoting palliative care principles in its city-wide operations (https://phpci.info/cities). Most of these cities employ, or are guided by, the Compassionate City Charter and this charter has an explicit policy-building agenda [20]. Perhaps in the future when these social experiments are more widely published, viewed comparatively with the more local compassionate communities studies, the balance between policy-building emphasis and the development of individual skills and knowledge may be better assessed.

A number of barriers and facilitators have been reported in the literature to guide compassionate community development and implementation. The published literature is also rich in examples of how to overcome common barriers, such as building awareness, adapting compassionate communities to diverse cultural settings, and building balanced and sustainable partnerships with community organizations. An apparent dissonance in findings is the emphasis on adaptation to local culture and social attitude (reported as the most common barrier to implementation) and the relative lack of compassionate community initiatives specifically targeting marginalized populations. Furthermore, the published literature places a heavy emphasis on the implementation and result stages, as opposed to the early phases of compassionate community development (stakeholder mobilization, needs assessment and priority-setting). This observation reflects a broader trend in the literature, which tends to view community development as a process of implementing pre-packaged interventions in the community, rather than a process of social empowerment driven and designed with the community [21]. Findings from the review points to a certain leadership style favoring the development of compassionate communities, where individuals and organizations share leadership and ownership of projects.

A minority of articles evaluated compassionate community initiatives, despite our restriction to peer-review publications. Research designs were largely non-comparative. When they are conducted, the vast majority of evaluations assess change at the individual level, as opposed to community and population change. This gap in evaluation is significant, given the role that evaluation can play in the development, improvement and sustainability of compassionate communities. The individual-level evaluation focus is congruent with pressures on community-based programs to demonstrate impact on individual health outcomes [22], difficulties in operationalizing ecological evaluation models [13], and the need for flexible approaches to evaluation that allows definition of compassionate communities’ goals by community members themselves [23, 24].

### Strengths, limitations and future research

This scoping review has provided a comprehensive overview of compassionate community initiatives in four important ways: 1- by describing the current pattern of participation, leadership, and practice emphasized in compassionate community programs in English-speaking countries; 2- by charting the growing profile, strength, and resulting influence of the compassionate communities movement in the health sciences literature in general, palliative the end-of-life care literature specifically; 3- by providing a state of the art understanding of the current challenges, barriers, and problems encountered in the implementation and research evaluation of these health promotion projects, and; 4- by providing a clearer academic assessment of the limitations of current research communications about this topic.

Because this has been a review of the English language peer-review literature, it is limited by its exclusion of non-English language publications. It has also been limited by excluding books, book chapters, grey literature reports, and unpublished compassionate community initiatives. These restrictions have three important implications for limiting our generalizations.

First, the culture-specific academic strategy of privileging journal literature in the biomedical and health sciences marginalizes the work of community activists, social sciences, and social care academics and workers who often employ other types of outputs, including books, to communicate their work. For example, Wegleitner, Heimerl and Kellehear (2016) document 10 empirical case studies of how different national examples of compassionate communities were implemented, but these cases appear in an edited book [25].

Secondly, many compassionate communities have been established in non-English-speaking countries and their evaluations are poorly represented in the English language literature, especially those from Europe, South America, South-East Asia, East Asia, and Africa. The existing English-language conceptual literature, especially those emerging from Taiwanese, Spanish, German, or Dutch writers for example, indicates major civic and academic activity around compassionate communities work, but this is largely inaccessible to English language-reviews. Furthermore, there are translation challenges even in English-language contexts. For example, in many German-speaking countries, compassionate communities are known as ‘caring communities’ [25, 26]. In Spanish-speaking contexts, where the term ‘compassion’ can have religious connotations or can have a closer and less desirable association with the word ‘pity’, the alternative phrases of ‘togetherness’ or ‘everybody’ in community has commonly been used. This means that even when translated into English, such community development examples would not necessarily be selected by the usual search terms employing community initiatives linked to the word ‘compassion’.

Thirdly, given the importance of developing countries and other areas of the world in the compassionate community movement, restriction to the peer-review literature could overemphasize the relative importance of professionally-led and academically-funded compassionate community initiatives, as opposed to grassroot community-led initiatives that may not have the resources (or priorities) to report their findings in the academic literature.

Finally, scoping reviews aim at mapping the existing literature rather than providing a synthesis of evidence about the effectiveness of specific interventions [10]. While our findings are useful to describe the range of available strategies used to develop, implement and evaluate compassionate communities, it cannot provides simple answers as to “what works best”.

Future research should seek to document emerging community-led initiatives being conducted outside of professional and academic settings, which may not be self-labeled as “compassionate communities” but nonetheless fall within the principles of health promoting palliative care. Future reviews should expand on geographical scope, language and cultural settings in which compassionate communities have been implemented. Early stages of compassionate community development, including engagement of community members in leadership and priority-setting stages, requires more research attention, as does the condition for adaptation to local culture, partnerships with marginalized communities and issues of sustainability.

Further research on the implementation and evaluation of compassionate communities might also include (even comparatively) the dementia-friendly communities’ movement [27]. Dementia-friendly communities—in terms of conceptual design and practice vision – are closely aligned with health promotion and civic program developments in end-of-life care. However, diagnosis-specific their origins have been compared to compassionate community programs, those programs remain part of the recent emergence, and merging, of ‘new’ public health ideas into health service management programs at the end-of-life.

Finally, it might also be useful to investigate and describe an additional and complementary scoping review that surveyed only the book, conference, or grey literature for their descriptions of implementation and evaluation of compassionate communities. This would provide a more interdisciplinary, non-health services research approach to this emerging field, and therefore a more complete portrayal. This would enlarge and deepen our understanding of an important end-of-life care initiative that has been embraced by the health and social care sectors, but also the broader civic society that is increasingly being asked to partner and support both.

### Implications for practice and policy

This review has important and useful implications for practice and policy. First, it lists a broad list of published examples of compassionate communities, using a variety of practical approaches and health promotion strategies. This is helpful to provide “grounding” and anchoring of the complex concepts of health promotion palliative and its translation into concrete examples and activities. The categorization of activities undertaken as part of compassionate communities’ implementation is an added value of this review, and could help practitioners reflect on a menu of possible strategies and ideas that may be adapted to their specific context. Identifying the main barriers and facilitators encountered on the ground may also help community members prospectively reflect on strategies to minimize them. Our findings also support the “slow growth” of compassionate community initiatives, highlighting the early stages of community mobilization, needs assessment and prioritization. The current global pandemic, with its heavy toll on grief, bereavement and social isolation, have further highlighted the importance and need for supportive networks among community members [28].

## Conclusion

The empirical literature on compassionate communities demonstrates a wide variety of health promotion practices across the world. Much international experience has been developed in education and awareness programs on death and dying. Health promotion strategies based on community strengthening and policies need to consolidated. Future research should pay attention to community-led initiatives and outcomes that may not be currently reported in the peer-review literature.

## Supplementary Information

**Additional file 1.** Search strategy. Describes exhaustively the search strategy (concepts, keywords and electronic databases).

**Additional file 2.** Description of included studies. Includes the titles of the selected studies, years, countries, population types and contexts.

**Additional file 3.** List of activities. Includes the activities and their categorization (development stages, implementation types, health promotion strategies) for each article reporting implemented activities in practice.

**Additional file 4.** Evaluation approaches. Includes detailed description of evaluation approaches (what is measured, how and at which levels) for each article reporting evaluation methods and results.

**Additional file 5.** Preferred Reporting Items for Systematic reviews and Meta-Analyses extension for Scoping Reviews (PRISMA-ScR) Checklist

## Data Availability

Original data is available upon request from the authors.
