# Supplementary material for: How compassionate communities are implemented and evaluated in practice: a scoping review
Source: BMC Palliat Care. 2022 Jul 20;21:131. doi: 10.1186/s12904-022-01021-3 (PMC9297657; doi:10.1186/s12904-022-01021-3)
Supplement: Supplementary file 1 — Additional file 1. Search strategy. Describes exhaustively the search strategy (concepts, keywords and electronic databases). [file 12904_2022_1021_MOESM1_ESM.docx]

***Search strategy***

Describes exhaustively the search strategy

**How compassionate communities are implemented and evaluated in practice: a scoping review**

Health and Social Services Systems, Knowledge Translation and Implementation component of the Quebec SPOR-SUPPORT Unit

May 13^th^, 2019

Nathalie Rheault, M.S.I., Information Specialist

Hervé Zomahoun, PhD

Agustina Gancia, Project Coordinator

Émilie Warren, Research Assistant

(concepts, keywords and electronic databases)


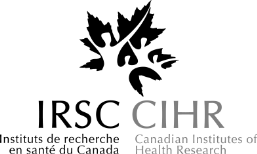


Table

[Search strategy 3](#_heading=h.30j0zll)

[Ovid Medline (2019-05-13) 4](#_heading=h.1fob9te)

[EBSCOhost-Cinahl (2019-05-13) 7](#_heading=h.3znysh7)

[Ovid PsycInfo (2019-05-13) 10](#_heading=h.2et92p0)

[Embase (2019-05-13) 13](#_heading=h.tyjcwt)

[Web of Science (2019-05-13) 15](#_heading=h.3dy6vkm)

[Consulted References 16](#_heading=h.1t3h5sf)

### Search strategy

**Research question:** How compassionate communities are implemented and evaluated in practice

**Population :** People receiving palliative care

**Intervention :** Compassionate Communities (All health promotion projects applied to end-of-life care)

**Comparison :** Any

**Outcomes :** Any

**Settings :** Any

**Study designs :** Any

**Databases**

1. Medline
2. Cinahl
3. PsycInfo
4. Embase
5. Web of Science

### Ovid Medline (2019-05-13)

| **PICOSS** | **Concepts** | **Search strategy keywords** | **Number** | **# Results** |
| --- | --- | --- | --- | --- |
| Population | Palliative Care (Controlled vocabulary) | exp Palliative Care/ or exp "Hospice and Palliative Care Nursing"/ or exp Terminal Care/ or exp Terminally Ill/ or exp Advance Care Planning/ | #1 | 96 077 |
|  | Palliative Care (Free text) | ( palliat* or ((dying or "end of life" or "end stag*" or endstag* or "eol" or "life-limiting illness" or terminal* or bereavement) adj6 (care or caring or comfort or intervention or plan or relief or strateg* or support*)) or "advance care planning" or "advance directive*" or "advanced care planning" or "advanced directive*" or "living will" ).ti.  or (palliat* or ((dying or "end of life" or "end stag*" or endstag* or "eol" or "life-limiting illness" or terminal* or bereavement) adj6 (care or caring or comfort or intervention or plan or relief or strateg* or support*)) or "advance care planning" or "advance directive*" or "advanced care planning" or "advanced directive*" or "living will").ab.  or (palliat* or ((dying or "end of life" or "end stag*" or endstag* or "eol" or "life-limiting illness" or terminal* or bereavement) adj6 (care or caring or comfort or intervention or plan or relief or strateg* or support*)) or "advance care planning" or "advance directive*" or "advanced care planning" or "advanced directive*" or "living will").kf. | #2 | 90 978 |
|  | Palliative Care | 1 or 2 | #3 | 141 293 |
| Intervention | Health Promotion (Controlled vocabulary) | exp Health Promotion/ | #4 | 72 055 |
|  | Community (Controlled vocabulary) | Cities/ | #5 | 16 448 |
|  | Community Health promotion projects | 4 and 5 | #6 | 189 |
|  | Health Promotion (Controlled vocabulary) | exp Health Promotion/ | #7 | 72 055 |
|  | Community (Free text) | (circle* or city or cities or civic or collectiv* or communit* or neighbo* or public* or rural or societ* or town* or urban* or village*).ti.  or (circle* or city or cities or civic or collectiv* or communit* or neighbo* or public* or rural or societ* or town* or urban* or village*).ab.  or (circle* or city or cities or civic or collectiv* or communit* or neighbo* or public* or rural or societ* or town* or urban* or village*).kf. | #8 | 1 700 925 |
|  | Community Health promotion projects | 7 and 8 | #9 | 25 995 |
|  | Community Health promotion projects (Controlled vocabulary) | exp Community Networks/ or exp Volunteers/ or Community Participation/ | #10 | 46 719 |
|  | Community Health promotion projects (Free text) | ((circle* or city or cities or civic or collectiv* or communit* or neighbo* or public* or rural or societ* or town* or urban* or village* or volunteer* or "voluntary worker*") adj2 (caregiving or caring or compassi* or cooperat* or empower* or engag* or "health promoti*" or involv* or mobilis* or mobiliz* or participat* or partnership* or support* or network*)).ti.  or ((circle* or city or cities or civic or collectiv* or communit* or neighbo* or public* or rural or societ* or town* or urban* or village* or volunteer* or "voluntary worker*") adj2 (caregiving or caring or compassi* or cooperat* or empower* or engag* or "health promoti*" or involv* or mobilis* or mobiliz* or participat* or partnership* or support* or network*)).ab.  or ((circle* or city or cities or civic or collectiv* or communit* or neighbo* or public* or rural or societ* or town* or urban* or village* or volunteer* or "voluntary worker*") adj2 (caregiving or caring or compassi* or cooperat* or empower* or engag* or "health promoti*" or involv* or mobilis* or mobiliz* or participat* or partnership* or support* or network*)).kf. | #11 | 53 468 |
|  | Community Health promotion projects | 10 or 11 | #12 | 94 320 |
|  | Community Health Promotion Projets (Total) | 6 or 9 or 12 | #13 | 115 344 |
|  | Total result | 3 AND 13 | #14 | 1 334 |
|  | Filter for human | exp Animals/ NOT exp Humans/ | #15 | 4 578 616 |
|  | With filter for human | 14 NOT 15 | #16 | 1 334 |

|  |  |
| --- | --- |

### EBSCOhost-Cinahl (2019-05-13)

| **PICOSS** | **Concepts** | **Search strategy keywords** | **Number** | **# Results** |
| --- | --- | --- | --- | --- |
| Population | Palliative Care (Controlled vocabulary) | (MH "Palliative Care") or (MH "Hospice and Palliative Nursing") or (MH "Terminal Care+") or (MH "Terminally Ill Patients+") or (MH "Advance Care Planning") **or** (MH "Dying Care (Iowa NIC)") or (MH "Bereavement Support (Saba CCC)") | #1 | 64 915 |
|  | Palliative Care (Free text) | TI ( palliat* or ((dying or "end of life" or "end stag*" or endstag* or "eol" or "life-limiting illness" or terminal* or bereavement) N5 (care or caring or comfort or intervention or plan or relief or strateg* or support*)) or "advance care planning" or "advance directive*" or "advanced care planning" or "advanced directive*" or "living will" )  OR AB ( palliat* or ((dying or "end of life" or "end stag*" or endstag* or "eol" or "life-limiting illness" or terminal* or bereavement) N5 (care or caring or comfort or intervention or plan or relief or strateg* or support*)) or "advance care planning" or "advance directive*" or "advanced care planning" or "advanced directive*" or "living will" )  OR SU ( palliat* or ((dying or "end of life" or "end stag*" or endstag* or "eol" or "life-limiting illness" or terminal* or bereavement) N5 (care or caring or comfort or intervention or plan or relief or strateg* or support*)) or "advance care planning" or "advance directive*" or "advanced care planning" or "advanced directive*" or "living will" ) | #2 | 66 397 |
|  | Palliative Care | S1 or S2 | #3 | 80 167 |
| Intervention | Health Promotion (Controlled vocabulary) | (MH "Health Promotion (Saba CCC)") OR (MH "Health Promotion") | #4 | 55 605 |
|  | Community (Controlled vocabulary) | (MH "Communities+") | #5 | 42 362 |
|  | Community Health promotion projects | S4 and S5 | #6 | 1 905 |
|  | Health Promotion (Controlled vocabulary) | (MH "Health Promotion (Saba CCC)") OR (MH "Health Promotion") | #7 | 55 605 |
|  | Community (Free text) | TI ( circle* or city or cities or civic or collectiv* or communit* or neighbo* or public* or rural or societ* or town* or urban* or village* )  OR AB ( circle* or city or cities or civic or collectiv* or communit* or neighbo* or public* or rural or societ* or town* or urban* or village* )  OR SU ( circle* or city or cities or civic or collectiv* or communit* or neighbo* or public* or rural or societ* or town* or urban* or village* ) | #8 | 779 185 |
|  | Community Health promotion projects | S7 and S8 | #9 | 21 598 |
|  | Community Health promotion projects (Controlled vocabulary) | (MH "Community Networks") or (MH "Volunteer Workers") or (MH "Volunteer Experiences") | #10 | 15 942 |
|  | Community Health promotion projects (Free text) | TI ( (circle* or city or cities or civic or collectiv* or communit* or neighbo* or public* or rural or societ* or town* or urban* or village* or volunteer* or "voluntary worker*") N1 (caregiving or caring or compassi* or cooperat* or empower* or engag* or "health promoti*" or involv* or mobilis* or mobiliz* or participat* or partnership* or support* or network*) )  OR AB ( (circle* or city or cities or civic or collectiv* or communit* or neighbo* or public* or rural or societ* or town* or urban* or village* or volunteer* or "voluntary worker*") N1 (caregiving or caring or compassi* or cooperat* or empower* or engag* or "health promoti*" or involv* or mobilis* or mobiliz* or participat* or partnership* or support* or network*) )  OR SU ( (circle* or city or cities or civic or collectiv* or communit* or neighbo* or public* or rural or societ* or town* or urban* or village* or volunteer* or "voluntary worker*") N1 (caregiving or caring or compassi* or cooperat* or empower* or engag* or "health promoti*" or involv* or mobilis* or mobiliz* or participat* or partnership* or support* or network*) ) | #11 | 27 832 |
|  | Community Health promotion projects | S10 or S11 | #12 | 40 740 |
|  | Community Health Promotion Projets (Total) | S6 or S9 or S12 | #13 | 59 629 |
|  | Total result | S3 AND S13 | #14 | 1 089 |

### Ovid PsycInfo (2019-05-13)

| **PICOSS** | **Concepts** | **Search strategy keywords** | **Number** | **# Results** |
| --- | --- | --- | --- | --- |
| Population | Palliative Care (Controlled vocabulary) | exp palliative care/ or exp advance directives/ or exp "death and dying"/ or exp terminally ill patients/ | #1 | 56 886 |
|  | Palliative Care (Free text) | (palliat* or ((dying or "end of life" or "end stag*" or endstag* or "eol" or "life-limiting illness" or terminal* or bereavement) adj6 (care or caring or comfort or intervention or plan or relief or strateg* or support*)) or "advance care planning" or "advance directive*" or "advanced care planning" or "advanced directive*" or "living will").ti.  or (palliat* or ((dying or "end of life" or "end stag*" or endstag* or "eol" or "life-limiting illness" or terminal* or bereavement) adj6 (care or caring or comfort or intervention or plan or relief or strateg* or support*)) or "advance care planning" or "advance directive*" or "advanced care planning" or "advanced directive*" or "living will").ab.  or (palliat* or ((dying or "end of life" or "end stag*" or endstag* or "eol" or "life-limiting illness" or terminal* or bereavement) adj6 (care or caring or comfort or intervention or plan or relief or strateg* or support*)) or "advance care planning" or "advance directive*" or "advanced care planning" or "advanced directive*" or "living will").hw. | #2 | 121 882 |
|  | Palliative Care | 1 or 2 | #3 | 122 561 |
| Intervention | Health Promotion (Controlled vocabulary) | exp health promotion/ | #4 | 72 055 |
|  | Community (Controlled vocabulary) | exp urban environments/ or exp communities/ or exp towns/ or exp rural environments/ | #5 | 172 854 |
|  | Community Health promotion projects | 4 and 5 | #6 | 2 875 |
|  | Health Promotion (Controlled vocabulary) | exp Health Promotion/ | #7 | 72 055 |
|  | Community (Free text) | (circle* or city or cities or civic or collectiv* or communit* or neighbo* or public* or rural or societ* or town* or urban* or village*).ti.  or (circle* or city or cities or civic or collectiv* or communit* or neighbo* or public* or rural or societ* or town* or urban* or village*).ab.  or (circle* or city or cities or civic or collectiv* or communit* or neighbo* or public* or rural or societ* or town* or urban* or village*).hw. | #8 | 1 971 383 |
|  | Community Health promotion projects | 7 and 8 | #9 | 31 005 |
|  | Community Health promotion projects (Controlled vocabulary) | exp community involvement/ or exp volunteers/ or exp social networks/ | #10 | 66 583 |
|  | Community Health promotion projects (Free text) | ((circle* or city or cities or civic or collectiv* or communit* or neighbo* or public* or rural or societ* or town* or urban* or village* or volunteer* or "voluntary worker*") adj2 (caregiving or caring or compassi* or cooperat* or empower* or engag* or "health promoti*" or involv* or mobilis* or mobiliz* or participat* or partnership* or support* or network*)).ti.  or ((circle* or city or cities or civic or collectiv* or communit* or neighbo* or public* or rural or societ* or town* or urban* or village* or volunteer* or "voluntary worker*") adj2 (caregiving or caring or compassi* or cooperat* or empower* or engag* or "health promoti*" or involv* or mobilis* or mobiliz* or participat* or partnership* or support* or network*)).ab.  or ((circle* or city or cities or civic or collectiv* or communit* or neighbo* or public* or rural or societ* or town* or urban* or village* or volunteer* or "voluntary worker*") adj2 (caregiving or caring or compassi* or cooperat* or empower* or engag* or "health promoti*" or involv* or mobilis* or mobiliz* or participat* or partnership* or support* or network*)).hw. | #11 | 71 901 |
|  | Community Health promotion projects | 10 or 11 | #12 | 120 414 |
|  | Community Health Promotion Projets (Total) | 6 or 9 or 12 | #13 | 146 149 |
|  | Total result | 3 AND 13 | #14 | 2 597 |

### Embase (2019-05-13)

| **PICOSS** | **Concepts** | **Search strategy keywords** | **Number** | **# Results** |
| --- | --- | --- | --- | --- |
| Population | Palliative Care (Controlled vocabulary) | 'palliative therapy'/exp OR 'palliative nursing'/exp OR 'terminal care'/exp or 'terminally ill patient'/exp OR 'advance care planning'/exp or 'living will'/exp OR 'dying'/exp OR 'end of life'/exp or 'bereavement support'/exp | #1 | 162 497 |
|  | Palliative Care (Free text) | (palliat* or ((dying or "end of life" or "end stag*" or endstag* or "eol" or "life-limiting illness" or terminal* or bereavement) NEAR/6 (care or caring or comfort or intervention or plan or relief or strateg* or support*)) or "advance care planning" or "advance directive*" or "advanced care planning" or "advanced directive*" or "living will"):ti,ab,kw | #2 | 137 001 |
|  | Palliative Care | #1 or #2 | #3 | 213 431 |
| Intervention | Health Promotion (Controlled vocabulary) | 'health promotion'/exp | #4 | 93 103 |
|  | Community (Controlled vocabulary) | 'city'/exp OR 'community'/exp OR 'community care'/exp OR 'neighborhood'/exp | #5 | 236 652 |
|  | Community Health promotion projects | #4 and #5 | #6 | 9 327 |
|  | Health Promotion (Controlled vocabulary) | 'health promotion'/exp | #7 | 93 103 |
|  | Community (Free text) | (circle* or city or cities or civic or collectiv* or communit* or neighbo* or public* or rural or societ* or town* or urban* or village*):ti,ab,kw | #8 | 3 054 744 |
|  | Community Health promotion projects | #7 and #8 | #9 | 34 513 |
|  | Community Health promotion projects (Controlled vocabulary) | 'community participation'/exp OR 'volunteer'/exp OR 'voluntary worker'/exp | #10 | 63 857 |
|  | Community Health promotion projects (Free text) | ((circle* or city or cities or civic or collectiv* or communit* or neighbo* or public* or rural or societ* or town* or urban* or village* or volunteer* or "voluntary worker*") NEAR/2 (caregiving or caring or compassi* or cooperat* or empower* or engag* or "health promoti*" or involv* or mobilis* or mobiliz* or participat* or partnership* or support* or network*)):ti,ab,kw | #11 | 66 440 |
|  | Community Health promotion projects | #10 or #11 | #12 | 126 530 |
|  | Community Health Promotion Projets (Total) | #6 or #9 or #12 | #13 | 158 374 |
|  | Total result | #3 AND #13 | #14 | 2 096 |
|  | Filter for human | 'animal'/exp not 'human'/exp | #15 | 5 242 391 |
|  | With filter for human | #14 NOT #15 | #16 | 2 103 |

|  |  |
| --- | --- |

### Web of Science (2019-05-13)

| **PICOSS** | **Concepts** | **Search strategy keywords** | **Number** | **# Results** |
| --- | --- | --- | --- | --- |
| Population | Palliative Care (Free text) | TS=(palliat* or ((dying or "end of life" or "end stag*" or endstag* or "eol" or "life-limiting illness" or terminal* or bereavement) NEAR/5 (care or caring or comfort or intervention or plan or relief or strateg* or support*)) or "advance care planning" or "advance directive*" or "advanced care planning" or "advanced directive*" or "living will") | #1 | 105 423 |
| Intervention | Community Health promotion projects (Free text) | TS=((circle* or city or cities or civic or collectiv* or communit* or neighbo* or public* or rural or societ* or town* or urban* or village* or volunteer* or "voluntary worker*") NEAR/1 (caregiving or caring or compassi* or cooperat* or empower* or engag* or "health promoti*" or involv* or mobilis* or mobiliz* or participat* or partnership* or support* or network*)) | #2 | 166 465 |
|  | Total result | 1 AND 2 | #3 | 1 720 |

|  |  |
| --- | --- |
